# Precise Expression of *Afmed15* Is Crucial for Asexual Development, Virulence, and Survival of Aspergillus fumigatus

**DOI:** 10.1128/mSphere.00771-20

**Published:** 2020-10-07

**Authors:** Luyu Guan, Ruiyang Lu, Zhengjun Wu, Guowei Zhong, Shizhu Zhang

**Affiliations:** a Jiangsu Key Laboratory for Microbes and Functional Genomics, Jiangsu Engineering and Technology Research Center for Microbiology, College of Life Sciences, Nanjing Normal University, Nanjing, China; b Key Laboratory of Ecology of Rare and Endangered Species and Environmental Protection (Guangxi Normal University), Ministry of Education, Guilin, China; c Guangxi Key Laboratory of Rare and Endangered Animal Ecology, Guangxi Normal University, Guilin, China; d Center for Global Health, School of Public Health, Nanjing Medical University, Nanjing, China; University of Georgia

**Keywords:** *Aspergillus fumigatus*, conidiation, fungal cell death, drug targets, inducible gene expression, opportunistic pathogen, *med15*

## Abstract

The rise of drug resistance in fungal pathogens is becoming a serious problem owing to the limited number of antifungal drugs available. Identifying and targeting factors essential for virulence or development unique to fungal pathogens is one approach to develop novel treatments for fungal infections. In this study, we present the identification and functional characterization of a novel developmental regulator in Aspergillus fumigatus, AfMed15, which contained a conserved Med15_fungal domain, as determined by screening of a mutant library that contained more than 2,000 hygromycin-resistant A. fumigatus transformants. Downregulating the expression of *Afmed15* abolished the conidiation and decreased the fungal virulence in an insect model. Strikingly, the overexpression of *Afmed15* caused fungal death accompanied by intensive autophagy. RNA sequencing of an *Afmed15* overexpression strain revealed that altered gene expression patterns were associated with carbon metabolism, energy metabolism, and translation. Interestingly, the addition of metal ions could partially rescue fungal death caused by the overexpression of *Afmed15*, indicating that disordered ion homeostasis is a potential reason for the fungal death caused by the overexpression of *Afmed15*. Considering that the precise expression of *Afmed15* is crucial for fungal development, virulence, and survival and that no ortholog was found in humans, *Afmed15* is an ideal target for antifungal-drug development.

**IMPORTANCE** The identification and characterization of regulators essential for virulence or development constitute one approach for antifungal drug development. In this study, we screened and functionally characterized *Afmed15*, a novel developmental regulator in A. fumigatus. We demonstrate that the precise transcriptional expression of *Afmed15* is crucial for fungal asexual development, virulence, and survival. Downregulating the expression of *Afmed15* abolished the conidiation and decreased the fungal virulence in an insect model. In contrast, the overexpression of *Afmed15* caused fungal death accompanied by intensive autophagy. Our study provides a foundation for further studies to identify compounds perturbing the expression of *Afmed15* that may be used for the prevention of invasive A. fumigatus infections.

## Abstract (precis)

The identification and characterization of regulators essential for virulence or development constitute one approach for antifungal drug development. In this study, we screened and functionally characterized *Afmed15*, a novel developmental regulator in A. fumigatus. We demonstrate that the precise transcriptional expression of *Afmed15* is crucial for fungal asexual development, virulence, and survival. Downregulating the expression of *Afmed15* abolished the conidiation and decreased the fungal virulence in an insect model. In contrast, the overexpression of *Afmed15* caused fungal death accompanied by intensive autophagy. Our study provides a foundation for further studies to identify compounds perturbing the expression of *Afmed15* that may be used for the prevention of invasive A. fumigatus infections.

## INTRODUCTION

Approximately 300 million people worldwide suffer from serious fungus-related diseases, and the occurrence of fungal infections has increased significantly in recent years owing to a rise in the number of immunocompromised patients (1, 2). Fungal infections are usually treated with antifungal drugs, such as polyenes, azoles, and echinocandins (3, 4). However, the rise of drug resistance in fungal pathogens is becoming a serious problem owing to the limited number of antifungal drugs available (5–7). Identifying and targeting factors essential for virulence or development that are unique to fungal pathogens constitute one approach for the development of novel treatments for fungal infections.

The filamentous fungus Aspergillus fumigatus is the most prevalent airborne fungal pathogen and causes severe invasive aspergillosis in immunocompromised patients (8). The majority of A. fumigatus strains are able to produce an enormous number of hydrophobic small asexual spores (conidia) (9). Humans are incidentally infected by the inhalation of small numbers of spores. In the absence of a well-balanced immune response, these conidia may germinate to form hyphae, which invade and destroy pulmonary tissue (10, 11). The central regulatory pathway that controls conidiation is highly conserved in *Aspergillus*. This pathway contains the key regulators BrlA, AbaA, and WetA, which coordinate conidiation-specific gene expression (12, 13). However, conidiation rarely occurs during invasive infection of the human host. In contrast, hyphae are the predominant fungal morphology observed during invasive pulmonary aspergillosis, while conidiation is rarely observed. However, more recently, it was proven that the dysregulation of the conidiation pathway via the overexpression of *brlA* reduced the vegetative growth of A. fumigatus *in vitro* and virulence *in vivo* (14). Therefore, a better understanding of the genetic regulatory mechanisms of fungal development will illuminate new approaches to control fungal disease.

Asexual development in *Aspergillus* also depends on autophagy, which is an evolutionarily conserved mechanism whereby cells recycle cellular elements, such as proteins and organelles, for degradation and recycling (15, 16). Autophagy plays diverse roles in medically important fungi. The deletion of the *atg1* gene, which encodes a serine/threonine kinase required for autophagy, caused abnormal conidiophore development and reduced conidiation in A. fumigatus (17). This result suggested that A. fumigatus uses autophagy to recycle internal resources to support the extensive remodeling that is required to complete conidiation. Despite a large number of reports on the requirement for autophagy in fungal differentiation and pathogenesis, the actual mechanistic role(s) of autophagy in medically important fungi remains largely unknown (16).

To identify new regulators in fungal development, we conducted a large-scale Agrobacterium tumefaciens-mediated transformant (ATMT) screen of A. fumigatus. A mutant library containing more than 2,000 hygromycin-resistant transformants of A. fumigatus was generated, and the mutants that exhibited asexual development defects were selected for further study. In this study, we present the identification and functional characterization of a novel regulator in A. fumigatus, encoded by *Afmed15*, which contains a conserved Med15_fungi domain. Lowering the expression of *Afmed15* abolished the conidiation and decreased the fungal virulence in an insect model. In contrast, the overexpression of *Afmed15* caused fungal death accompanied by intensive autophagy. Considering that the precise expression of *Afmed15* is crucial for fungal development, virulence, and survival and that no ortholog was found in humans, *Afmed15* would be an ideal target for antifungal-drug development.

## RESULTS

### Screening and identification of *Afmed15* as a developmental regulator in A. fumigatus.

**FIG 1:**
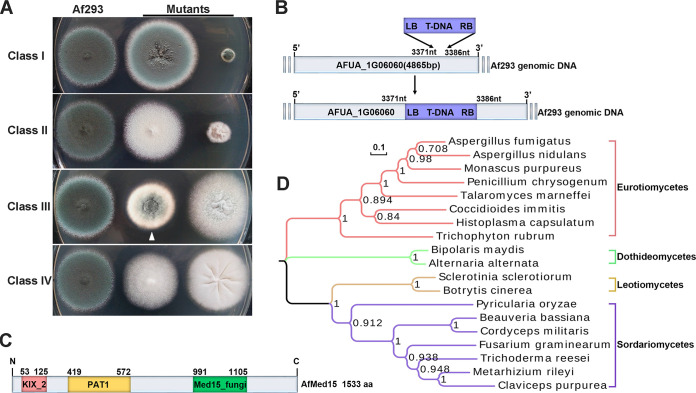
Identification of *Afmed15* through T-DNA insertion and distribution of Med15 proteins in fungi. (A) Comparison of reference (Af293) colonies and the representative colonies of T-DNA insertion mutants in four different categories grown on YAG agar plates. Plates were incubated at 37°C for 2 days. The T1033 mutant is indicated by an arrowhead. (B) Schematic of T-DNA insertion sites in the genomic regions of T1033 mutant. The insertion site determined by TAIL-PCR lies between nt 3371 and 3386 downstream of the translational start codon of the gene AFUA_1G06060. (C) Schematic diagram of conserved domains of AfMed15 in A. fumigatus. AfMed15 contains a KIX_2 domain, a PAT1 domain, and a Med15_fungi domain. (D) Phylogenetic analysis of AfMed15 orthologs for selected organisms represents the four different taxonomic classes within Pezizomycotina. Sequences were obtained from the NCBI protein database. Multiple-sequence alignment was performed by using MAFFT, and the phylogenetic tree was constructed by using MEGA-X maximum-likelihood analyses.

To identify novel fungal developmental regulators, a random transfer DNA (T-DNA) insertion mutant library that contained approximately 2,000 transformants of A. fumigatus (Af293) was constructed (5). A set of morphologic mutants was classified into four categories (Fig. 1A). In class I, the mutants showed accelerated or retarded hyphal growth but did not display any alterations in asexual development. In class II, the mutants produced a normal amount of conidia that were white. In classes III and IV, the mutants displayed reduced conidiation and nonconidiating phenotypes, respectively, compared with that of the wild type.

In this study, the insertional mutant T1033 (i.e., transformant 1033), which belonged to class III, showed a reduced capacity for conidiation and an impaired capacity for colony growth. The T-DNA flanking sequences in the T1033 mutant were successfully amplified using thermal asymmetric interlaced PCR (TAIL-PCR). This experiment demonstrated that the inserted T-DNA fragment lay between nucleotides 3371 and 3386 downstream of the translational start codon of gene AFUA_1G06060 (Fig. 1B). Protein analysis using SMART (http://smart.embl-heidelberg.de/) and the Conserved Domain Database of the NCBI (CDD) revealed that AFUA_1G06060 has three predicted domains, which include KIX_2 (an activator-binding domain; amino acids [aa] 53 to 125), PAT1 (topoisomerase II-associated protein; aa 419 to 572), and Med15_fungi (mediator complex subunit 15; aa 991 to 1105) (Fig. 1C). At this point, we designated this gene *Afmed15* and named its protein AfMed15, on the basis that it harbors the 115-aa-residue domain Med15_fungi. In addition, the real-time reverse transcription-PCR (RT‐PCR) analysis demonstrated that *Afmed15* expression was higher in asexual development and mature hyphae (24-h vegetative growth) stages than in the younger hyphae (12-h vegetative growth) (Fig. S1).

10.1128/mSphere.00771-20.1 FIG S1 Relative mRNA expression of *Afmed15* during A. fumigatus development as determined by RT-PCR. Results are means and standard deviations from three replicates (*n* = 3). Asterisks indicate significant differences from RNA expression levels in 12-h vegetative hyphae (**, *P* < 0.01; ****, *P* < 0.0001 [unpaired Student’s *t* test]). Download FIG S1, TIF file, 0.2 MB. Copyright © 2020 Guan et al. 2020 Guan et al. This content is distributed under the terms of the Creative Commons Attribution 4.0 International license.

The full-length sequence of AfMed15 was first used to search the A. fumigatus protein database in GenBank. This search showed that the A. fumigatus genome encodes only one copy of the AfMed15 protein. This sequence was then used to search for orthologs in fungi. The results revealed that AfMed15 orthologs are primarily present in four lineages: Sordariomycetes, Eurotiomycetes, Leotiomycetes, and Dothideomycetes in the Pezizomycotina. However, no orthologs of AfMed15 exist in the genomes of the ascomycetous yeasts Saccharomyces cerevisiae, Schizosaccharomyces pombe, and Candida albicans. Furthermore, the phylogenetic relationship among these orthologs for selected organisms was analyzed, and a phylogenetic tree was created using MEGA-X maximum-likelihood analyses (Fig. 1D). Med15 proteins tended to form four distinct clusters. Among them, AfMed15A shared the highest degree of homology (62%) with its corresponding ortholog in Aspergillus nidulans (locus_tag AN4210) and the lowest identity scores (19%) with its ortholog in Neurospora crassa (locus_tag NCU00124).

### *Afmed15* is involved in fungal asexual development and virulence.

**FIG 2:**
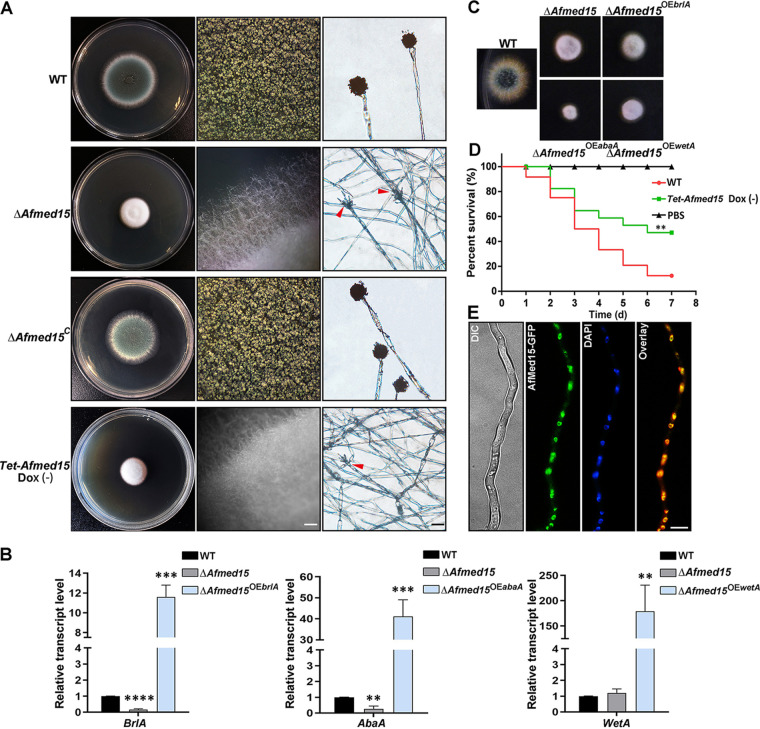
Phenotypic characterization of the Δ*Afmed15* mutant. (A) Colony morphology (left panels) of the reference strain A1161 and the Δ*Afmed15*, Δ*Afmed15^C^*, and *Tet-Afmed15* (OFF) mutants. The conidia were spotted on YUU plates at 37°C for 2 days. Individual colony pictures (middle panels) were taken under a dissecting microscope (bar, 200 μm). Micrographs of conidiophores (right panels) were also taken (bar, 20 μm). Arrowheads indicate conidiophores of mutants bearing no spore. (B) Expression analysis of *brlA*, *abaA*, and *wetA* genes by quantitative PCR in Δ*Afmed15*, Δ*Afmed15*^OE^*^brlA^*, Δ*Afmed15*^OE^*^abaA^*, and Δ*Afmed15*^OE^*^wetA^* mutants and reference strain A1161. Values are means and standard deviations (SD) from three independent experiments (**, *P* < 0.01; ***, *P* < 0.001; ****, *P* < 0.0001 [unpaired Student's *t* test]). (C) Colony phenotype comparison of wild-type, Δ*Afmed15*, Δ*Afmed15*^OE^*^brlA^*, Δ*Afmed15*^OE^*^abaA^*, and Δ*Afmed15*^OE^*^wetA^* strains. (D) The *Tet-Afmed15* (OFF) strain has attenuated virulence in the G. mellonella model (**, *P* < 0.01; *n* = 30). (E) Fluorescent microscopy of vegetative hyphae expressing Afmed15-green fluorescent protein (AfMed15-GFP) and counterstained with 4′,6-diamidine-2′-phenylindole dihydrochloride (DAPI). Bar, 10 μm.

To confirm that the phenotype of the T1033 mutant was affected by the *Afmed15* insertion mutation, we next constructed a mutant with full-length deletion of *Afmed15* in the background of strain A1160 (Fig. S2A). The Δ*Afmed15* mutant exhibited a reduced colony size similar to that of the T1033 mutant (Fig. 2A). Strikingly, conidiation was completely abolished in the Δ*Afmed15* mutant. The difference in conidiation between T1033 and the Δ*Afmed15* mutants may be caused by a remnant function of the truncated *Afmed15* fragment in this insertion mutant. In addition, the hyphal growth and conidiation defects of Δ*Afmed15* mutant were remediated by transformation with the full-length *Afmed15* gene (Fig. 2A). To investigate in more detail whether AfMed15 orthologs have a conserved function in *Aspergillus*, *Anmed15* (AN4210.4), an *AfMed15* ortholog in A. nidulans, was deleted, resulting in a Δ*Anmed15* strain. As shown in Fig. S2B, the Δ*Anmed15* strain displayed conidiation and hyphal defects as seen in the Δ*Afmed15* strain, indicating a conserved role for Med15 orthologs in hyphal growth and conidiation in *Aspergillus*.

10.1128/mSphere.00771-20.2 FIG S2 (A) PCR analysis showed that the full-length sequence of *Afmed15* was deleted in the Δ*Afmed15* mutant. For lanes 2 and 4, PCR was used to determine whether *Afmed15* still existed in the genome, and the expected size was 1.55 kb. For lanes 1 and 3, PCR was used to determine whether *Afmed15* was replaced by the auxotrophy gene *Ncpyr4* in the genome, and the expected size was 1.47 kb. (B) Colony phenotype comparison of the A. nidulans reference strain (TN02A7) and the Δ*Anmed15* strain. The indicated strains were tested on YUU plates and cultured at 37°C for 2 days. Download FIG S2, TIF file, 1.7 MB. Copyright © 2020 Guan et al. 2020 Guan et al. This content is distributed under the terms of the Creative Commons Attribution 4.0 International license.

Since the deletion of *Afmed15* abolished conidiation, we next examined if *Afmed15* regulates the expression of the *brlA*, *abaA*, and *wetA* pathway genes, which had been verified as central regulators of asexual development in *Aspergillus*. The mRNA levels of *brlA* and *abaA*, but not *wetA*, were significantly downregulated during the asexual developmental stage in the Δ*Afmed15* strain compared with the wild-type strain (Fig. 2B). We sought to determine whether the overexpression of these regulators was sufficient to restore conidiation in the Δ*Afmed15* strain. To this end, we constructed *brlA*, *abaA*, and *wetA* overexpression mutants (Δ*Afmed15*^OE^*^brlA^*, Δ*Afmed15*^OE^*^abaA^*, and Δ*Afmed15*^OE^*^wetA^*) in the Δ*Afmed15* background using the *gpdA* promoter. The levels of expression of *brlA*, *abaA*, and *wetA* in their corresponding overexpression strains were confirmed by quantitative reverse transcription-PCR (qRT-PCR) analysis (Fig. 2B). However, the overexpression of *brlA*, *abaA*, and *wetA* could not rescue the conidiation defect of Δ*Afmed15* (Fig. 2C).

To overcome the conidiation defect in the *Afmed15* deletion strain, an inducible *Afmed15* (*Tet-Afmed15*) strain was constructed, in which *Afmed15* was placed under the control of a doxycycline-inducible promoter (18). The integration site was confirmed by PCR (Fig. S3A). As expected, when cultured on rich YUU medium (see below), the *Tet-Afmed15* (OFF) strain recapitulated the radial growth defect and the nonconidiating phenotypes observed in the *Afmed15* deletion mutant (Fig. 2A). We further analyzed the effect of *Afmed15* in the wax moth Galleria mellonella. In this insect model, G. mellonella larvae infected with the *Tet-Afmed15* (OFF) strain showed a significantly higher survival rate than those infected with the wild type (WT) (*P* < 0.01) over a period of 7 days (Fig. 2D). These data suggest that Afmed15 is involved in fungal virulence. To gain insight into the subcellular location of AfMed15, we constructed a strain in which AfMed15 was labeled with green fluorescent protein (GFP) at the C terminus. This allows the GFP fusion target protein to be natively expressed under the control of its own promoter. By using fluorescence microscopy, live-cell imaging signals of GFP-AfMed15 were observed to exhibit a nuclear localization pattern in the hyphal cells (Fig. 2E). This suggests that AfMed15 functions predominantly in the nucleus. Collectively, the above results suggest that AfMed15 is a nuclear localization protein and plays an essential role in asexual development and virulence in *Aspergillus*.

10.1128/mSphere.00771-20.3 FIG S3 (A) Diagnostic PCR confirmed that the promoter of *Afmed15* was replaced by the pyrithiamine resistance gene in strain A1161. For lanes 1, 4, and 7 and lanes 3, 6, and 9, PCR primer pairs were tet-Afmed15 P1/tet-verification up and tet-verification down/tet-Afmed15 P6, to detect whether there was homologous recombination to replace the promoter of *Afmed15* with the pyrithiamine resistance gene at the 5′ and 3′ junctions, and the expected sizes were 1.7 kb and 3 kb, respectively; for lanes 2, 5, and 8, the PCR primer pair was tet-Afmed15-P SF/SR, to detect whether promoter of *Afmed15* still exists in the genome, and the expected size was 0.6 kb. (B) Comparison of colonies of parental wild-type strain A1161 and the *Tet*-*Afmed15* strain on YAG plates in the absence or presence of the indicated concentrations of doxycycline at 37°C for 2 days. Download FIG S3, TIF file, 2.3 MB. Copyright © 2020 Guan et al. 2020 Guan et al. This content is distributed under the terms of the Creative Commons Attribution 4.0 International license.

### Overexpression of *Afmed15* caused fungal cell death.

**FIG 3:**
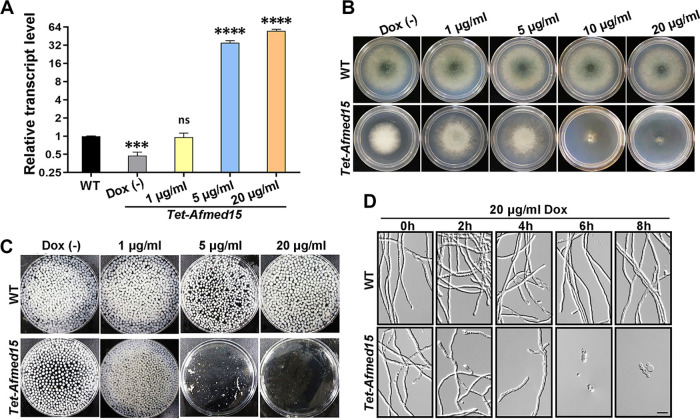
Overexpression of *Afmed15* causes fungal cell death. (A) Expression analysis of *Afmed15* by quantitative PCR of the *Tet-Afmed15* strain with various concentrations of doxycycline. Values are means and SD from three independent experiments (***, *P* < 0.001; ****, *P* < 0.0001; ns, not significant [unpaired Student's *t* test]). (B) Comparison of colonies of parental wild-type A1161 and *Tet-Afmed15* strains on MM plates in the absence or presence of the indicated concentrations of doxycycline at 37°C for 48 h. (C) Biomass assay of parental wild-type A1161 and *Tet-Afmed15* strains on liquid MM in the absence or presence of the indicated concentrations of doxycycline at 37°C for 24 h. (D) The wild-type A1161 and *Tet-Afmed15* strains were first cultured on MM in the presence of 20 μg/ml doxycycline for 2 h, 4 h, 6 h, and 8 h, and then the doxycycline-pretreated strains were cultured for a further 12 h on MM without doxycycline. Germination was observed under the microscope (bar, 10 μm).

Consistent with previous reports (14), doxycycline treatment of the *Tet-Afmed15* mutant resulted in dose-dependent expression of *Afmed15* in this strain. The expression of *Afmed15* was reduced by 50% compared with the parental wild-type in the absence of doxycycline (OFF). *Afmed15* was expressed as highly as the wild type in the presence of 1 μg/ml doxycycline. In comparison, *Afmed15* was overexpressed 30- and 60-fold in the presence of 5 μg/ml and 20 μg/ml doxycycline, respectively, compared with the wild type (Fig. 3A). As expected, the *Tet-Afmed15* (OFF) strain recapitulated the radial growth defect and the nonconidiating phenotypes when cultured on minimal medium (MM), as observed in the *Afmed15* deletion mutant. The addition of 1 μg/ml of doxycycline to MM resulted in hyphal radial growth of *Tet-Afmed15* (ON) to the wild-type level. In comparison, the conidiation defect was partially rescued under the same condition. Interestingly, the hyphal growth of *Tet-Afmed15* was greatly inhibited when it was cultured on MM supplemented with doxycycline at concentrations higher than 5 μg/ml (OE) (Fig. 3B). Consistently, the biomass of the *Tet-med15* strain was almost abolished when it was exposed to 5 μg/ml or 20 μg/ml of doxycycline on MM (Fig. 3C). Similar results were obtained when the *Tet-Afmed15* strain was cultured on YAG rich medium with a series of concentrations of doxycycline, except that better conidiation was observed in the presence of 1 μg/ml doxycycline, although it still did not reach the wild-type level (Fig. S3B).

To further confirm if the overexpression of *Afmed15* inhibited fungal growth or caused fungal death, we first incubated the *Tet-Afmed15* strain in the presence of 20 μg/ml doxycycline for 2, 4, 6, and 8 h and then cultured the doxycycline-pretreated strain for an additional 12 h on MM without doxycycline. The germination rate was less than 5% when the *Afmed15* strain was pretreated for 6 h with 20 μg/ml doxycycline (Fig. 3D). This result suggested that the overexpression of *Afmed15* caused fungal cell death instead of inhibiting fungal growth.

### Overexpression of *Afmed15* caused intensive autophagy.

**FIG 4:**
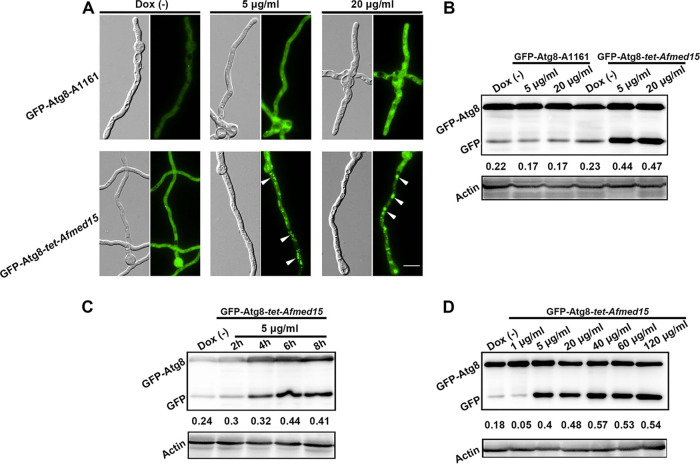
Overexpression of *Afmed15* caused intensive autophagy. (A) Epifluorescence micrographs of autophagosomes. Mutants expressing the GFP-Atg8 fusion gene were grown in liquid MM for 8 h and then cultured for a further 8 h in the presence of 5 μg/ml and 20 μg/ml doxycycline. As controls, the same strains were cultured in liquid MM in the absence of doxycycline for 12 h. Arrowheads indicate autophagosomes. Bar, 10 μm. (B) Immunoblot analysis of GFP-Atg8 proteolysis. Mutants expressing the GFP-Atg8 fusion gene were grown in liquid MM for 16 h and then cultured for a further 8 h in the presence of 5 μg/ml and 20 μg/ml doxycycline. As controls, the same strains were cultured in liquid MM in the absence of doxycycline for 24 h. The numbers below the Western blot indicate the quantified intensity of GFP/(GFP-Atg8 plus GFP) ratios. (C) Doxycycline treatment resulted in time-dependent autophagy. A GFP-Atg8-expressing Tet-Afmed15 strain was grown in liquid MM for 16 h and then cultured for a further 2 h, 4 h, 6 h, and 8 h in the presence of 5 μg/ml doxycycline. The level of autophagy was detected by Western blotting, employing a GFP-specific antibody. (D) Doxycycline resulted in dose-dependent autophagy. A GFP-Atg8-expressing Tet-Afmed15 strain was grown in liquid MM for 16 h and then cultured for a further 8 h in the presence of 5 μg/ml, 20 μg/ml, 40 μg/ml, 60 μg/ml, and 120 μg/ml doxycycline. The level of autophagy was detected by Western blotting, employing a GFP-specific antibody.

Autophagy and apoptosis are commonly accompanied by cell death. To determine whether apoptosis or autophagy accounted for the loss of viability in *Afmed15*-overexpressing cells, we examined the cells for markers of autophagy and apoptosis. First, we introduced the autophagy marker fusion gene encoding GFP-Atg8 into the *Tet-Afmed15* and wild-type strains for autophagic flux analysis using epifluorescence microscopy and Western blotting (19). The wild-type strain contained very few autophagosomes, and little GFP-Atg8 fluorescence was observed within the hyphal cytoplasm when the strain was grown in MM with and without doxycycline. In contrast, the *Tet-Afmed15* mutant accumulated more autophagosomes and exhibited strong GFP-Atg8 fluorescence inside the cytoplasm in the presence of 5 and 20 μg/ml doxycycline (Fig. 4A). We further assessed the autophagic flux by analyzing vacuolar delivery and the subsequent breakdown of GFP-Atg8. The wild-type strain contained smaller amounts of free GFP than GFP-Atg8 in the presence or absence of doxycycline, suggesting that the wild-type strain had a relatively low level of autophagic flux. In contrast, the *Tet-Afmed15* mutant accumulated larger amounts of free GFP in the presence of 5 μg/ml and 20 μg/ml doxycycline but not in the absence of doxycycline, indicating an increase in autophagic flux as a consequence of the overexpression of *Afmed15* (Fig. 4B). Furthermore, doxycycline treatment of the *Tet-Afmed15* mutant resulted in time-dependent autophagy in this strain. At least 4 h was needed to cause autophagy in the *Tet-Afmed15* mutant when it was exposed to 5 μg/ml of doxycycline (Fig. 4C). Moreover, the levels of autophagic flux respond in a doxycycline dose-dependent manner, in which 40 μg/ml of doxycycline induced higher levels of autophagic flux than 5 μg/ml of doxycycline (Fig. 4D).

We sought to confirm whether high levels of autophagy are the reason for cell death. We hypothesized that blocking autophagy would increase the cell survival rate when *Afmed15* was overexpressed. Autophagy requires a unique set of factors called autophagy-related (Atg) proteins. Among them, Atg2 is important for autophagosome formation (20). As shown in Fig. S4A, the deletion of *atg2* in the *Tet-Afmed15* mutant could not restore the hyphal growth of *Tet-Afmed15* mutant in the presence of 10 μg/ml or 20 μg/ml of doxycycline. These results suggest that elevated autophagy is not the cause of the growth defect that results from *Afmed15* overexpression.

10.1128/mSphere.00771-20.4 FIG S4 (A) Colony morphology of *Tet-Afmed15*, *Tet-Afmed15*^OE^*^bir1^*, and Δ*atg2-GFP-ATG8-Tet-Afmed15* strains in the presence of 0, 1, 10, and 20 μg/ml of doxycycline. Strains were tested on MM plates and cultured at 37°C for 2 days. (B) Observation of caspase activity among wild-type and *Tet*-*Afmed15* strains. The indicated strains were cultured in liquid MM for 8 h and exposed to the indicated treatments for a further 6 h (bar, 10 μm). Download FIG S4, TIF file, 1.9 MB. Copyright © 2020 Guan et al. 2020 Guan et al. This content is distributed under the terms of the Creative Commons Attribution 4.0 International license.

We also examined cells for markers of apoptosis via the detection of caspase activity of the *Tet-Afmed15* mutant by staining with a fluorescein isothiocyanate (FITC)-labeled VAD-fmk probe, which has a high binding affinity for caspase (21). In contrast with H_2_O_2_ treatment, no increase in the intensity of fluorescence of FITC was observed when *Afmed15* was overexpressed (Fig. S4B). In addition, we overexpressed A. fumigatus *bir1* in the *Tet*-*Afmed15* mutant. The A. fumigatus *bir1* protein is a homolog of human survivin (22) and S. cerevisiae Bir1 (23), both of which are inhibitors of apoptosis protein family members. In A. fumigatus, the overexpression of *bir1* can reduce apoptosis-like programmed cell death caused by oxidative stress (21). However, the overexpression of A. fumigatus *bir1* did not restore the hyphal growth of *Tet-Afmed15* in the presence of 10 μg/ml or 20 μg/ml doxycycline (Fig. S4A). Collectively, the results indicated that the cell death caused by the overexpression of *Afmed15* involved autophagy. However, it appears that autophagy and apoptosis are not the major reasons for fungal death when *Afmed15* was overexpressed.

### RNA sequencing of *Afmed15* overexpression reveals altered gene expression patterns associated with carbon metabolism, energy metabolism, and translation.

**FIG 5:**
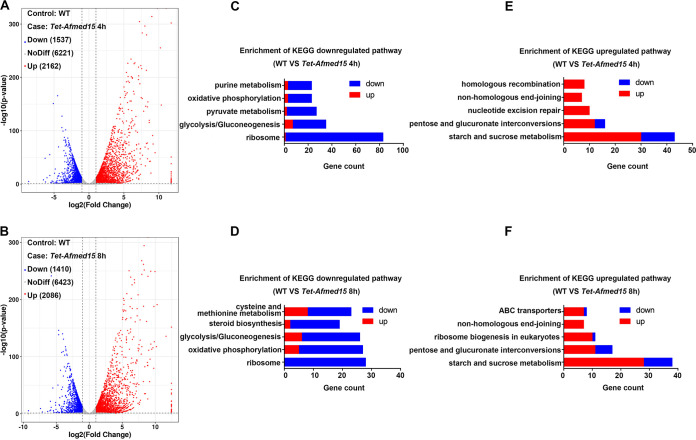
High-level *Afmed15* overexpression results in significant changes in the patterns of gene transcription. (A and B) Volcano plot highlighting downregulated and upregulated genes with a |(log_2_FC)| of >1 and a *P* value of <0.05. Differential gene expression in the *Tet-Afmed15* strain exposed to 5 μg/ml doxycycline for 4 h (A) and 8 h (B) compared with the parental wild type was measured by RNA sequencing. (C and D) Enrichment analysis of genes downregulated in the *Tet-Afmed15* strain exposed to 5 μg/ml doxycycline for 4 h (C) and 8 h (D) compared with the parental wild type using the Kyoto Encyclopedia of Genes and Genomes (KEGG) catalogued pathways for A. fumigatus. (E and F) Enrichment analysis of genes upregulated in the *Tet-Afmed15* strain exposed to 5 μg/ml doxycycline for 4 h (E) and 8 h (F) compared with the parental wild type using KEGG catalogued pathways for A. fumigatus.

To explore the mechanisms by which a high level of expression of *Afmed15* mediates fungal cell death, a transcriptome sequencing (RNA-seq)-based approach was used. Two groups of samples were collected. In group 1, conidia from the *Tet-Afmed15* mutant were cultured on MM for 20 h and then exposed to 5 μg/ml doxycycline for an additional 4 h before RNA extraction (*Tet-Afmed15* OE 4). In group 2, conidia from the *Tet-Afmed15* mutant were cultured on MM for 16 h and then exposed to 5 μg/ml doxycycline for an additional 8 h before RNA extraction (*Tet-Afmed15* OE 8). Conidia of the wild type that were cultured on MM for 24 h served as the control. The profile of gene expression in the *Tet-Afmed15* mutant was strongly affected by the overexpression of *Afmed15*. A total of 2,162 genes were upregulated and 1,537 genes were downregulated using a |(log_2_FC)| (where FC is fold change) cutoff value of >1 in the *Tet-Afmed15* OE 4 group compared with the wild-type control (Fig. 5A). In comparison, 2,086 genes were upregulated and 1,410 genes downregulated in the *Tet-Afmed15* OE 8 group compared with the wild type (Fig. 5B).

A gene set enrichment analysis was performed to identify the pathways most affected by the high-level overexpression of *Afmed15*. A list of differentially regulated genes (FC > 2, *P* < 0.05) in the doxycycline-treated *Tet-Afmed15* mutant was compared to Kyoto Encyclopedia of Genes and Genomes (KEGG) catalogued pathways. The representative categories of downregulated genes when *Afmed15* was overexpressed included the ribosome, glycolysis/gluconeogenesis, and oxidative phosphorylation pathways. The results indicated that the overexpression of *Afmed15* caused the processes of translation, carbohydrate metabolism, and energy metabolism to be greatly reduced (Fig. 5C and D). In contrast, the representative categories of upregulated genes were exclusively enriched in the processes of metabolism, including starch and sucrose metabolism, pentose and glucuronate interconversions, and nonhomologous end-joining (Fig. 5E and F). Taken as a whole, these findings suggest that the high-level overexpression of *Afmed15* reversed the carbon flux, decreased the energy metabolism, and shut down the process of translation.

### Addition of metal ions could partially rescue the fungal cell death caused by the overexpression of *Afmed15*.

**FIG 6:**
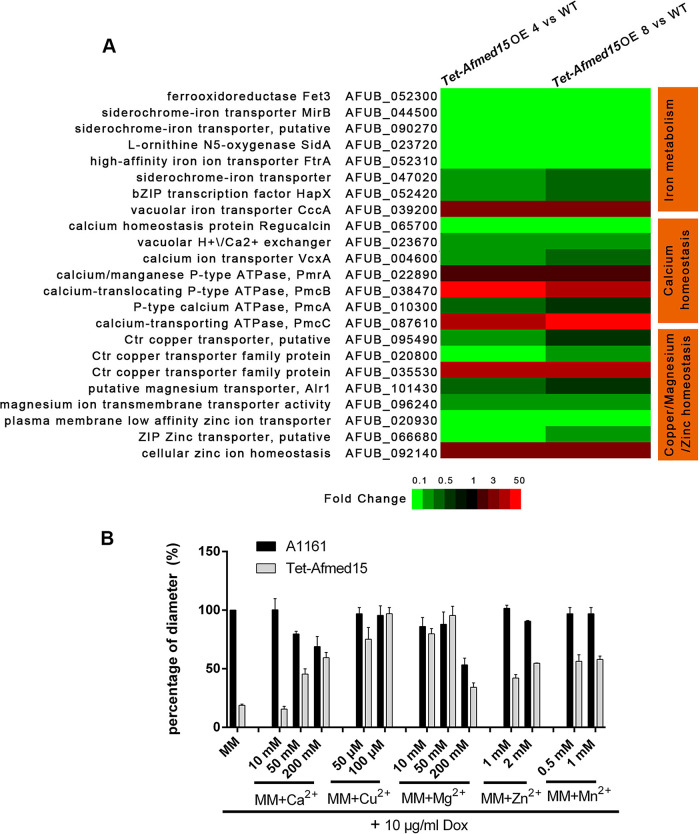
Addition of metal ions partially rescued fungal cell death caused by overexpression of *Afmed15*. (A) Heat map analysis of RNA-seq results for the annotated genes encoding proteins putatively involved in iron, calcium, zinc, manganese, magnesium, and copper uptake and storage. (B) Colony sizes, shown as percentages of the diameter of the control strain (A1161), for the indicated strains cultured on MM plates with a series of concentrations of metal ions in the presence of 10 μg/ml doxycycline.

The results of RNA-seq also showed that the overexpression of *Afmed15* resulted in the altered expression of genes involved in the uptake and storage of divalent cations, such as calcium, zinc, manganese, iron, magnesium, and copper (Fig. 6A). Thus, we tested if the addition of metal ions could restore hyphal growth when *Afmed15* was overexpressed in the *Tet-Afmed15* mutant. As shown in Fig. 6B, the addition of 50 mM or 200 mM calcium, 50 μM or 100 μM copper, 10 mM or 50 mM magnesium, 1 mM or 2 mM zinc, and 0.5 mM or 1 mM manganese to MM dramatically promoted the hyphal growth of the *Tet-Afmed15* strain in the presence of 10 μg/ml doxycycline. In contrast, no obvious conidiation was observed under the same conditions (Fig. S5A).

10.1128/mSphere.00771-20.5 FIG S5 Colony morphology of parental wild type and *Tet-Afmed15* strains. Two microliters of conidial suspension containing 1 × 10^4^, 1 × 10^3^, or 1 × 10^2^ conidia of each strain was used to inoculate MM with a series of concentrations of metal ions in the presence (A) and absence (B) of 10 μg/ml doxycycline. Download FIG S5, TIF file, 2.4 MB. Copyright © 2020 Guan et al. 2020 Guan et al. This content is distributed under the terms of the Creative Commons Attribution 4.0 International license.

We also tested the effect of metal ions on the *Tet-Afmed15* (OFF) mutant. The addition of 50 mM calcium, 2 mM zinc, or 1 mM manganese promoted hyphal growth. In contrast, the metal ions did not suppress the nonconidiating phenotype of the *Tet-Afmed15* (OFF) strain (Fig. S5B). Collectively, these results indicated that disordered ion homeostasis is a potential reason for the fungal death caused by the overexpression of *Afmed15* but not the major reason for conidiation defect when *Afmed15* was turned off.

## DISCUSSION

In this study, we identified a novel fungal developmental regulator, *Afmed15*, using T-DNA library screening. Moreover, we demonstrated that the precise expression of *Afmed15* is important for fungal asexual development, virulence, and survival. Downregulating the expression of *Afmed15* abolished the production of conidia associated with the decreased expression of the asexual developmental master regulators *brlA* and *abaA*. However, overexpression of *brlA* and *abaA* did not rescue the conidiation defect phenotype of the *Tet-Afmed15* strain. This indicated that Afmed15 has additional roles in conidiation beyond regulation of *brlA* and *abaA*.

An attractive finding in this study is that overexpression of *Afmed15* led to fungal cell death. A variety of environmental stimuli, including multiple drugs, as well as small antimicrobial proteins produced by microorganisms, animals, humans, and plants, can lead to fungal programmed cell death (24–26). Recently, it was reported that overexpression of *brlA* during vegetative growth could activate the conidiation pathway in A. fumigatus and inhibit vegetative growth. However, it is not clear if this inhibition could lead to cell death (14). The results of our RNA-seq studies provide evidence for the *Afmed15*-dependent dysregulation of the carbon flux, in which the genes involved in glycolysis/gluconeogenesis and pyruvate metabolism were greatly downregulated when *Afmed15* was overexpressed. In contrast, the genes involved in starch and sucrose metabolism and pentose and glucuronate interconversions were upregulated under the same conditions. In addition, energy metabolism was reduced when *Afmed15* was overexpressed, which led to substantial downregulation of the genes involved in the oxidative phosphorylation pathway. Moreover, the ribosome biogenesis and translation genes were greatly repressed, and almost all the KEGG enriched genes involved in the ribosome were downregulated.

Furthermore, we found that the overexpression of *Afmed15* was accompanied by intensive autophagy. The degradative functions of autophagy contribute to several important aspects of cell physiology, including autophagy-dependent programmed cell death. For example, the rice blast fungus Magnaporthe grisea undergoes a regulated form of programmed cell death during appressorium development that involves autophagy (27). Thus, we hypothesized that the overexpression of *Afmed15* caused autophagy-dependent fungal death. However, the possibility still exists that autophagy is activated in a failed effort to mitigate cell damage in which the inhibition of autophagy promotes rather than protects against cell death (28, 29). To distinguish between these hypotheses, we blocked autophagy by deleting *atg2* in the *Tet*-*Afmed15* mutant. The result showed that the fungal death caused by overexpression of *Afmed15* was not rescued by the deletion of *atg2*. This finding suggested that the cell death caused by overexpression of *Afmed15* involved autophagy. However, it appears that autophagy is not the major cause of fungal death when *Afmed15* is overexpressed. In addition, the activity of caspase was not detected when *Afmed15* was overexpressed. Moreover, the overexpression of the gene *bir1*, which encodes an inhibitor of apoptosis, could not rescue the cell death caused by the overexpression of *Afmed15*. These results exclude apoptosis from the process of overexpression of *Afmed15* that results in fungal death.

Interestingly, we found that the addition of metal ions, such as calcium, zinc, manganese, magnesium, and copper, could partially restore the growth of the *Tet-Afmed15* mutant when *Afmed15* was overexpressed. These results suggest that *Afmed15* contributes to the maintenance of metal ion homeostasis. Indeed, the overexpression of *Afmed15* caused the dysregulated expression of genes involved in uptake and storage of metal ions, including calcium, zinc, manganese, iron, magnesium, and copper. Using calcium homeostasis as an example, the expression of *pmcB* (AFUB_038470) and *pmcC* (AFUB_087610) was upregulated 20- to 50-fold when *Afmed15* was overexpressed. In contrast, the expression of *pmcA* (AFUB_010300) slightly decreased (approximately 0.5-fold) under the same conditions. *pmcA* to *pmcC* encode putative vacuolar Ca^2+^ ATPases that are involved in depleting the cytosol of Ca^2+^ ions (30–32). Fungal vacuolar Ca^2+^ ATPases are involved in removing Ca^2+^ ions from the cytosol and transporting them to internal stores, thus avoiding calcium toxicity. The overexpression of *pmcB* and *pmcC* suggested that more Ca^2+^ ions from the cytosol move to internal stores. In addition, the expression of *pmrA* (AFUB_022890) was also upregulated when *Afmed15* was overexpressed. PmrA is a high-affinity Ca^2+^/Mn^2+^ P-type ATPase required for Ca^2+^ and Mn^2+^ transport into the Golgi body (33, 34). However, the expression of *vcxA* (AFUB_004600) and its homolog (AFUB_023670) was decreased. In S. cerevisiae, VCX1 is a vacuolar membrane antiporter with Ca^2+^/H^+^ and K^+^/H^+^ exchange activity that is involved in the control of cytosolic concentrations of Ca^2+^ and K^+^ (30). In addition, the genes involved in zinc, manganese, iron, magnesium, and copper uptake and storage were also subjected to dysregulation. The result exhibited that the distribution and storage of ion were dysfunctional when *Afmed15* was overexpressed, and it also linked ion homeostasis and fungal cell death.

Collectively, our study identified a novel fungal developmental regulator, *Afmed15*, and provides a foundation for additional studies to identify compounds perturbing the expression of *Afmed15* that may be used for the prevention of invasive A. fumigatus infections.

## MATERIALS AND METHODS

### Strains, media, and culture conditions.

The strains of *Aspergillus* used in this study are listed in Table S1. The media used in this study included YAG (2% glucose, 0.5% yeast extract, and trace elements), YUU (YAG supplemented with 5 mM uridine and 10 mM uracil), and MM (1% glucose, 70 mM NaNO_3_, trace elements, and salts) (35). To induce the expression of *Afmed15* at different levels in the *Tet-Afmed15* mutant, the indicated concentrations of doxycycline were added to the medium.

10.1128/mSphere.00771-20.6 TABLE S1 Aspergillus strains used in this study. Download Table S1, DOCX file, 0.02 MB. Copyright © 2020 Guan et al. 2020 Guan et al. This content is distributed under the terms of the Creative Commons Attribution 4.0 International license.

### Construction and screening of the T-DNA random insertional mutant library of A. fumigatus.

*Agrobacterium*-mediated transformation was conducted as previously described (36). In brief, conidia of A. fumigatus 293 and A. tumefaciens strain EHA105 were cocultivated at a ratio of 1:10 (conidia to bacteria) on induction medium supplemented with 200 μM acetosyringone, a phenolic compound that induces T-DNA to enter the recipient strain. After cocultivation for 48 h at 24°C, YAG medium supplemented with hygromycin (300 μg/ml) and cefotaxime (200 μg/ml) was used to select the transformants.

### Cloning of unknown flanking sequences.

To ascertain the T-DNA insertion sites, TAIL-PCR was performed as previously described (37). TAIL-PCR is commonly composed of three nested amplifications. The primers used in each amplification reaction consisted of left or right border primers, corresponding to the border sequence of the T-DNA, and an AD primer (Table S2). All TAIL-2 and TAIL-3 products were sequenced, and the resulting sequences were then used as queries to perform a BLAST analysis in the A. fumigatus database.

10.1128/mSphere.00771-20.7 TABLE S2 Primers used in this study. Download Table S2, DOCX file, 0.02 MB. Copyright © 2020 Guan et al. 2020 Guan et al. This content is distributed under the terms of the Creative Commons Attribution 4.0 International license.

### Construction of *Afmed15* and *Anmed15* gene deletion and complementary strains.

Fusion PCR was used to construct the *Afmed15* knockout cassette as previously described (38). In brief, approximately 1-kb sections of regions flanking the *Afmed15* gene were amplified using the primers Afmed15 P1/P3 and Afmed15 P4/P6. The selection marker *pyr4* from the plasmid pAL5 was amplified with the primers Pyr4 F/R. Next, the three PCR products were used as the template to generate the *Afmed15* deletion cassette using the primers Afmed15 P2/P5 and then transformed into the parental A. fumigatus strain A1160 as previously described (39). Transformants were verified by diagnostic PCR using the primers Afmed15 SF/SR, Afmed15 P1/Pyr4 down, and Pyr4 up/Afmed15 P6, respectively. A similar strategy was used to construct the *Anmed15* knockout cassette by using primers Anmed15 P1/P3 for the 5′ region, Anmed15 P4/P6 for the 3′ region, Pyr4 F/R for selection marker *pyr4*, and Afmed15 P2/P5 for the fusion product. The final cassette was purified and used to transform A. nidulans strain TN02A7. Transformants were verified by diagnostic PCR using primers Anmed15 SF/SR, Anmed15 P1/Pyr4 down, and Pyr4 up/Anmed15 P6, respectively.

To construct the Δ*Afmed15* complemented strain, a PCR-generated DNA fragment including the *Afmed15* open reading frame (ORF) plus approximately 1 kb upstream of ATG and 1 kb downstream of the stop codon was obtained using primers Afmed15-up-XbaI and Afmed15-down-HindIII. This fragment was subsequently cloned into the XbaI and HindIII site of the pAN7-1 plasmid, which contains the hygromycin B resistance gene *hph*, to generate the *Afmed15* complementation plasmid Afmed15-com-hph. The plasmid was then transformed into the *Afmed15* deletion strain, and transformants were selected on YAG medium supplemented with 200 μg/ml hygromycin. The primers used in this study are shown in Table S2.

### Overexpression of the *brlA*, *abaA*, and *wetA* genes in the Δ*Afmed15* mutant.

To overexpress the *brlA* gene in the Δ*Afmed15* mutant background, the hygromycin B resistance gene *hph* was amplified with the primers hph-up-SpeI and hph-down-SpeI and then cloned into the SpeI site of pBARGPE to generate pBARGPE-hph. The ORF of *brlA* was amplified from the genomic DNA of A1160 with the primers gpd-BrlA F and gpd-BrlA R and then subcloned into the EcoRI site of pBARGPE-*hph* to generate a *brlA* overexpression plasmid, OEBrlA-hph. OEBrlA-hph was randomly integrated into the genome of the Δ*Afmed15* mutant to obtain the Δ*Afmed15*^OE^*^brlA^* strain. An identical strategy was used to obtain the Δ*Afmed15*^OE^*^abaA^* and Δ*Afmed15*^OE^*^wetA^* strains.

### Construction of the AfMed15-GFP strain.

An AfMed15-GFP fusion cassette was constructed as previously described (38). Briefly, a *gfp*-*pyrG* fragment was amplified from plasmid pFNO3 using the primer pair GFPPyrG F/R. An approximately 1-kb fragment immediately upstream of the *Afmed15* stop codon and a 1-kb fragment immediately downstream of the *Afmed15* stop codon were amplified using the primer pairs Afmed15GFP P1/P3 and Afmed15GFP P4/P6, respectively. These fragments were fused by PCR using primers Afmed15GFP P2/P5, and the PCR product was used to transform strain A1160. Homologous integration was verified by PCR using the primers Afmed15GFP P1/Pyr4 R and GFPPyr4 F/Afmed15GFP P6, respectively.

### Fluorescence microscopy.

To visualize the localization of Afmed15, the AfMed15-GFP strain was grown on coverslips in YAG medium at 37°C for 10 h. To stain nuclei, 4′,6-diamidine-2′-phenylindole dihydrochloride (DAPI) dissolved in phosphate-buffered saline (PBS) was used at a final concentration of 0.5 μg/ml and incubated for 30 min at the room temperature after the cells had been fixed with 4% paraformaldehyde. Images were captured using a Zeiss Axio imager A1 microscope (Zeiss, Jena, Germany), and the picture was managed with Adobe Photoshop.

### Construction of the *Tet-Afmed15* strain.

To overcome the conidiation defect in the *Afmed15* deletion mutant, a strain with conditional expression of *Afmed15* was generated. First, the pyrithiamine resistance cassette and the Tet-On system were amplified from plasmid pCH008 using the primer pair Tet-Afmed15 SF/SR. A fragment of approximately 1 kb immediately upstream of ATG and a 1-kb fragment immediately downstream of ATG of *Afmed15* were amplified using the primer pairs tet-fmed15 P1/P3 and tet-Afmed15 P4/P6, respectively. These fragments were fused by PCR using the primers tet-Afmed15 P2/P5, and the PCR product was used to transform strain A1161. Homologous integration was verified by PCR using the primers tet-Afmed15 P1/tet-verification up and tet-verification down/tet-Afmed15 P6, respectively.

### Generation of a mutant expressing the GFP-Atg8 fusion protein.

To monitor the autophagic process in A. fumigatus, the GFP-Atg8 strain, in which Atg8 was labeled with GFP at the N terminus under the control of the A. nidulans *gpdA* (*AngpdA*) promoter, was generated as previously described (19). Briefly, a GFP-Atg8 fragment was amplified from the plasmid gpdA(p)-GFP-Atg8 using the primer pair Gpd-GFP-Atg8-F/R. Subsequently, this fragment was cloned to the XbaI and HindIII site of the plasmid pAN7-1, which contained the hygromycin B resistance gene (*hph*), to generate the plasmid GFP-Atg8-hph. GFP-Atg8-hph was ectopically integrated into the genome of A1161 to generate the strain GFP-Atg8-A1161. To obtain strain GFP-Atg8-tet-Afmed15, the aforementioned *Tet*-*afmed15* cassette was transformed into GFP-Atg8-A1161. Homologous integration was verified as described above.

### Construction of the Δatg2-GFP-Atg8-A1161 and Δatg2-GFP-Atg8-Tet-Afmed15 strains.

To construct the *atg2* knockout cassette, approximately 1-kb sections of the flanking regions of the *atg2* gene were amplified using primers Δatg2 P1/P3 and Δatg2 P4/P6. The selection marker *phle* from the plasmid pTATA was amplified with the primers Phle F/R. Next, the three PCR products were used as the template to generate the *atg2* deletion cassette using the primers atg2 P2/P5. The resultant PCR product was then transformed into the strains GFP-Atg8-A1161 and GFP-Atg8-tet-Afmed15. Transformants were verified by diagnostic PCR using the primers atg2 SF/SR, atg2 P1/Phle down, and Phle up/atg2 P6.

### Overexpression of the *bir1* gene in the *Tet*-*Afmed15* strain.

The ORF of *bir1* was amplified from the genomic DNA of A1160 with primers gpd-BIR1 F and gpd-BIR1 R and then subcloned into the EcoRI site of pBARGPE-*hph* to generate a *birA* overexpression plasmid, OEbir1-hph. The plasmid OEbir1-hph was transformed into the *Tet*-*Afmed15* mutant to obtain the *Tet-Afmed15*^OE^*^bir1^* strain.

### G. mellonella virulence assay.

Virulence assays in G. mellonella were carried out as described by Fallon et al. (40), with some modifications. In a brief, G. mellonella larvae were injected through the hind prolegs with 10 μl of PBS containing 5 × 10^5^ conidia of the respective strain. Untreated larvae injected with 10 μl of PBS served as controls. Larvae were incubated at 37°C in the dark and monitored daily up to 7 days. Significance of survival data was evaluated by using Kaplan-Meier survival curves, analyzed with the log-rank (Mantel-Cox) test utilizing GraphPad Prism software.

### RNA extraction and qRT-PCR.

To detect the expression of *Afmed15* after treatment with various concentrations of doxycycline, the *Tet-Afmed15* strain was first cultured in MM medium for 16 h and then supplemented with the indicated concentrations of doxycycline for 8 h. To detect the expression of *brlA*, *abaA*, and *wetA* during the sporulation period of A. fumigatus, the relevant strains were first cultured in liquid MM for 24 h. They were then transferred to a solid plate of MM and cultured for 12 h. The total RNA was isolated using TRIzol following the manufacturer’s instructions. Genomic DNA digestion and cDNA synthesis were performed using a HiScript R II Q RT SuperMix kit for qPCR (Vazyme, Jiangsu, China) according to the manufacturer’s instructions. qRT-PCR was performed using an ABI One-step Fast thermocycler (Applied Biosystems, Foster City, CA, USA) with SYBR Premix Ex Taq (Vazyme). The results were then normalized to *tubA* and calculated using the ΔΔ*C_T_* method (41). All of the qRT-PCR primers are shown in Table S2.

### Transcriptional profile analyses using RNA-seq.

Two group of samples were collected for RNA sequencing. In group 1, conidia of the *Tet-Afmed15* mutant were cultured on MM for 20 h and then exposed to 5 μg/ml doxycycline for an additional 4 h before RNA extraction (*Tet-Afmed15* OE 4). In group 2, conidia of the *Tet-Afmed15* mutant were cultured on MM for 16 h and then exposed to 5 μg/ml doxycycline for an additional 8 h before RNA extraction (*Tet-Afmed15* OE 8). Conidia of the wild-type were cultured on MM for 24 h as a control. The samples were then collected and subsequently frozen in liquid nitrogen. The RNA isolation, mRNA purification and cDNA synthesis and sequencing were performed by Shanghai Personal Biotechnology (Shanghai, China). All the experiments were conducted in triplicate.

### Protein extraction and Western blotting.

For whole-cell lysate extraction, the mycelia were ground with liquid nitrogen and alkaline lysis buffer (0.2 M NaOH and 0.2% β-mercaptoethanol), and a mediated protein isolation strategy was followed as previously described (45). Briefly, 20 mg of powdered mycelium was suspended in 1 ml lysis buffer. A volume of 75 μl of trichloroacetic acid (TCA) was added, and the samples were vortexed and incubated on ice for 10 min. After centrifugation at 13,000 × *g* for 5 min at 4°C, the supernatants were removed. The pellets were heated to 95°C and vortexed in 100 μl of 1 M Tris and 100 μl of 2× SDS protein sample buffer until they had completely dissolved. For Western blot analysis, GFP and actin were detected using an anti-GFP mouse monoclonal antibody (catalog no. 11 814 460 001; Roche) and anti-actin antibody (clone C4; ICN Biomedical, Inc., Aurora, OH, USA), respectively. Western blotting was performed as previously described (42).

### Detection of fungal caspase activity.

The caspase *in situ* labeling fluorescence analysis system for the FITC–VAD-fmk probe (G7461; Promega Corp., Madison, WI, USA) was used to stain the activity of fungal caspase as previously described, with some modifications (43, 44). Briefly, approximately 400 to 500 μl of conidia of the *Tet-Afmed15* and wild-type strains suspended in liquid MM were adsorbed onto aseptic cover slides in small dishes and cultured at 37°C for 8 h. After one washing with PBS, the cells were exposed to MM with 20 μg/ml doxycycline or 5 mM H_2_O_2_ for an additional 6 h. After one washing with phosphate-buffered saline (PBS), the cells were stained with 200 μl staining solution containing 10 μM FITC–VAD-fmk. After incubation for 20 min at room temperature in the dark, the cells were washed twice with PBS and analyzed using fluorescence microscopy.

### Data availability.

The RNA-seq data have been deposited in the NCBI Sequence Read Archive with accession code PRJNA663649.
