# Supplementary figures and images for: Precise Expression of Afmed15 Is Crucial for Asexual Development, Virulence, and Survival of Aspergillus fumigatus
Source: mSphere. 2020 Oct 7;5(5):e00771-20. doi: 10.1128/mSphere.00771-20 (PMC7568654; doi:10.1128/mSphere.00771-20)

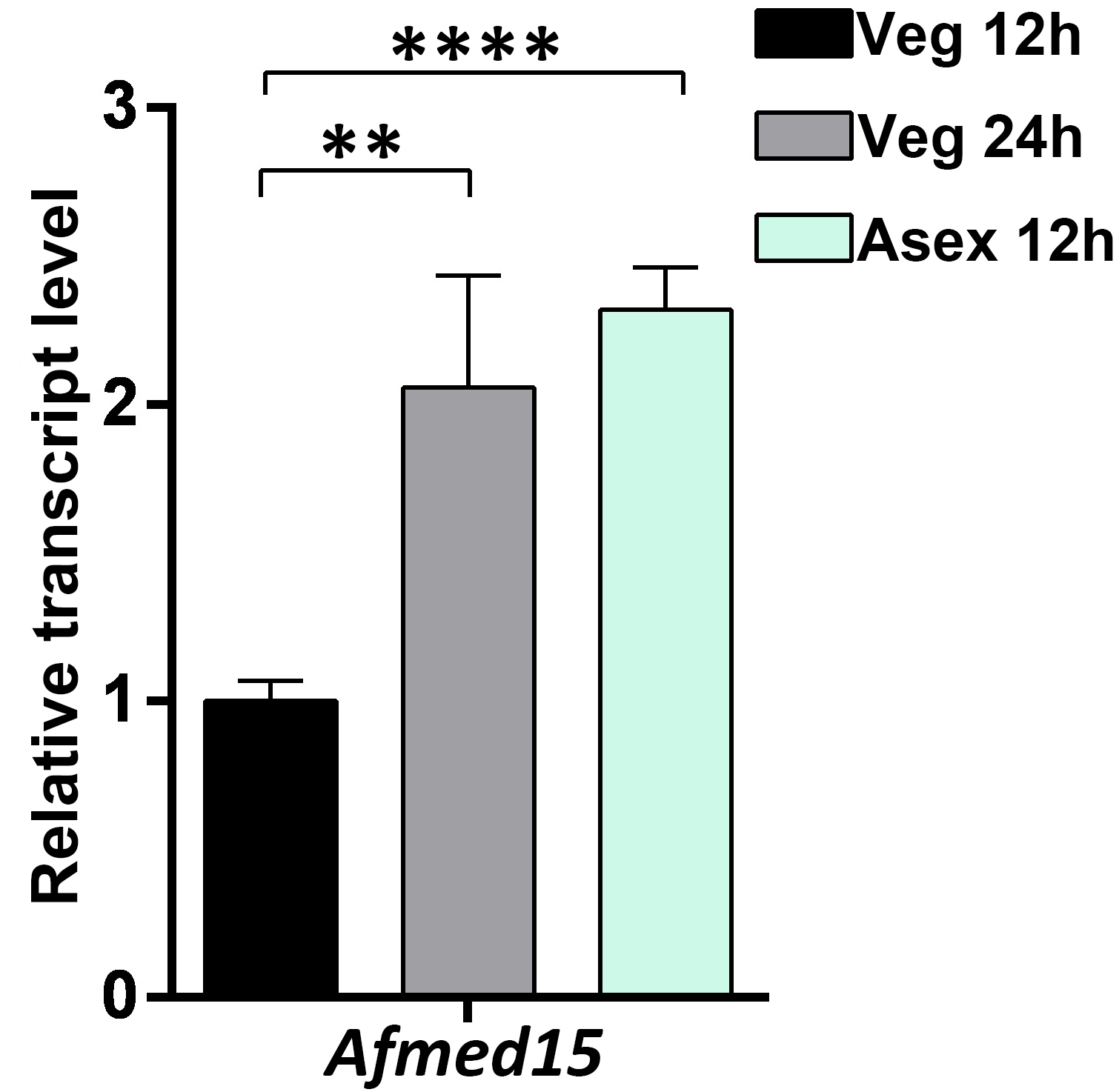

Supplement: FIG S1 [file mSphere.00771-20-sf001.tif]

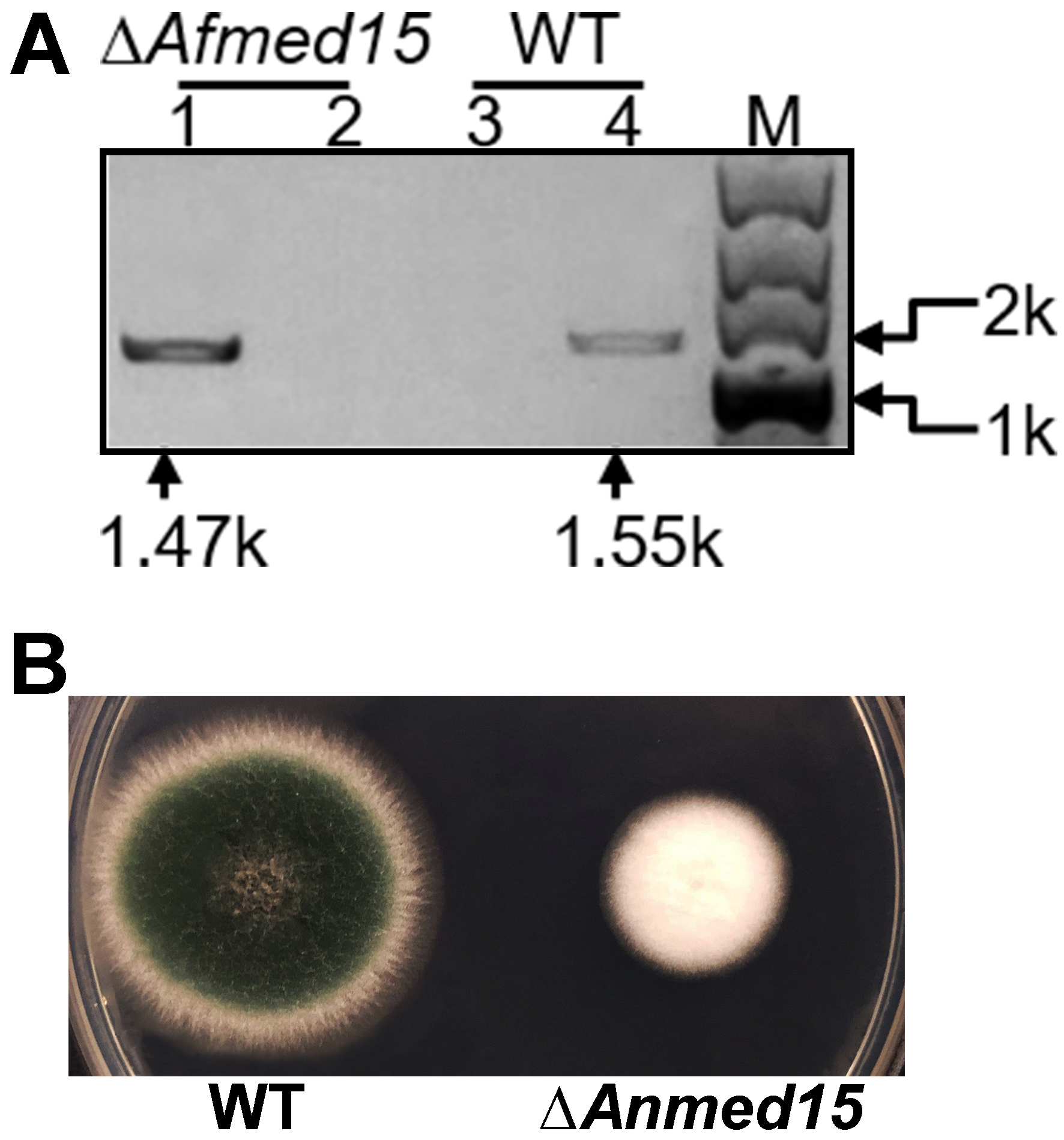

Supplement: FIG S2 [file mSphere.00771-20-sf002.tif]

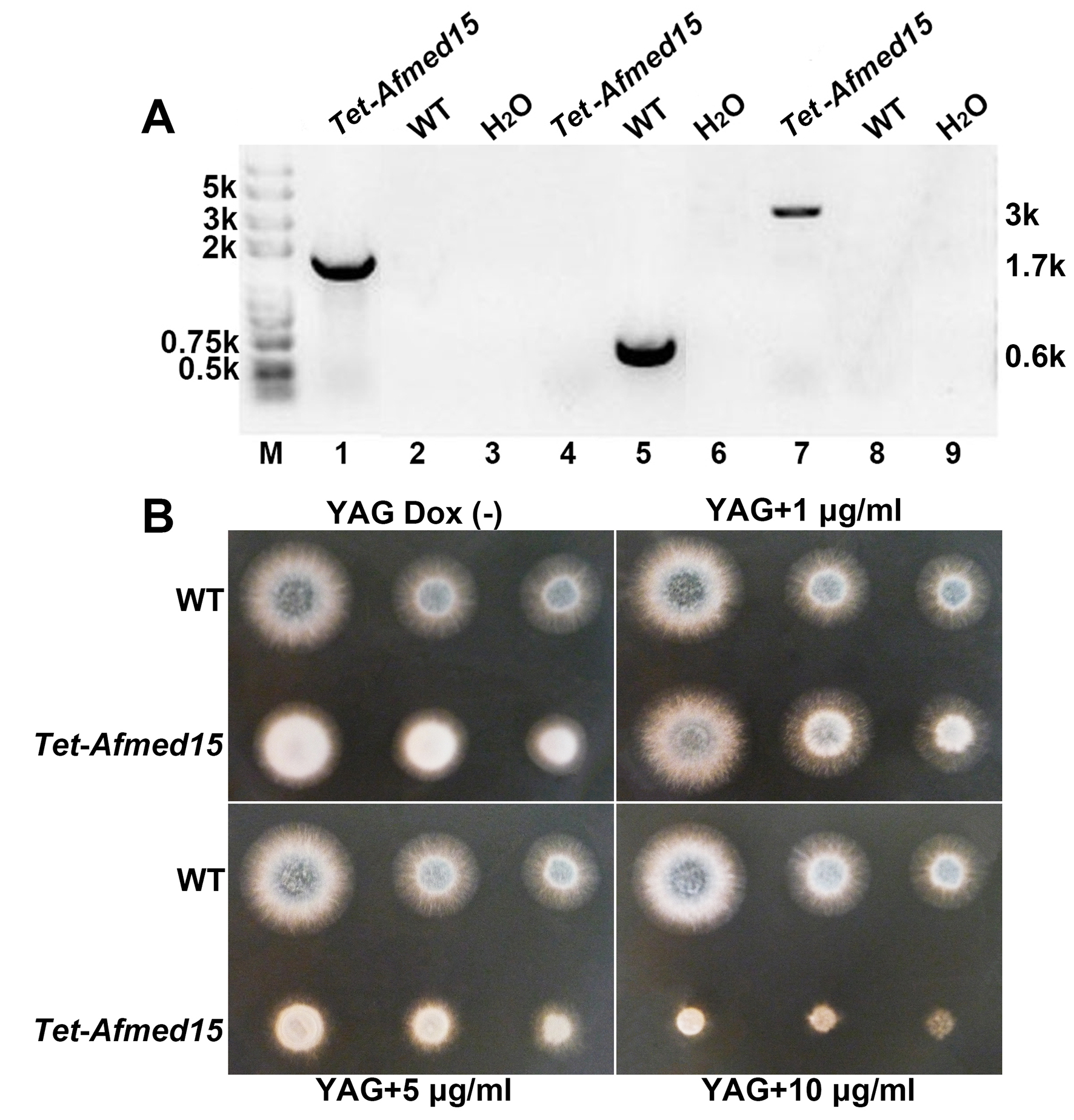

Supplement: FIG S3 [file mSphere.00771-20-sf003.tif]

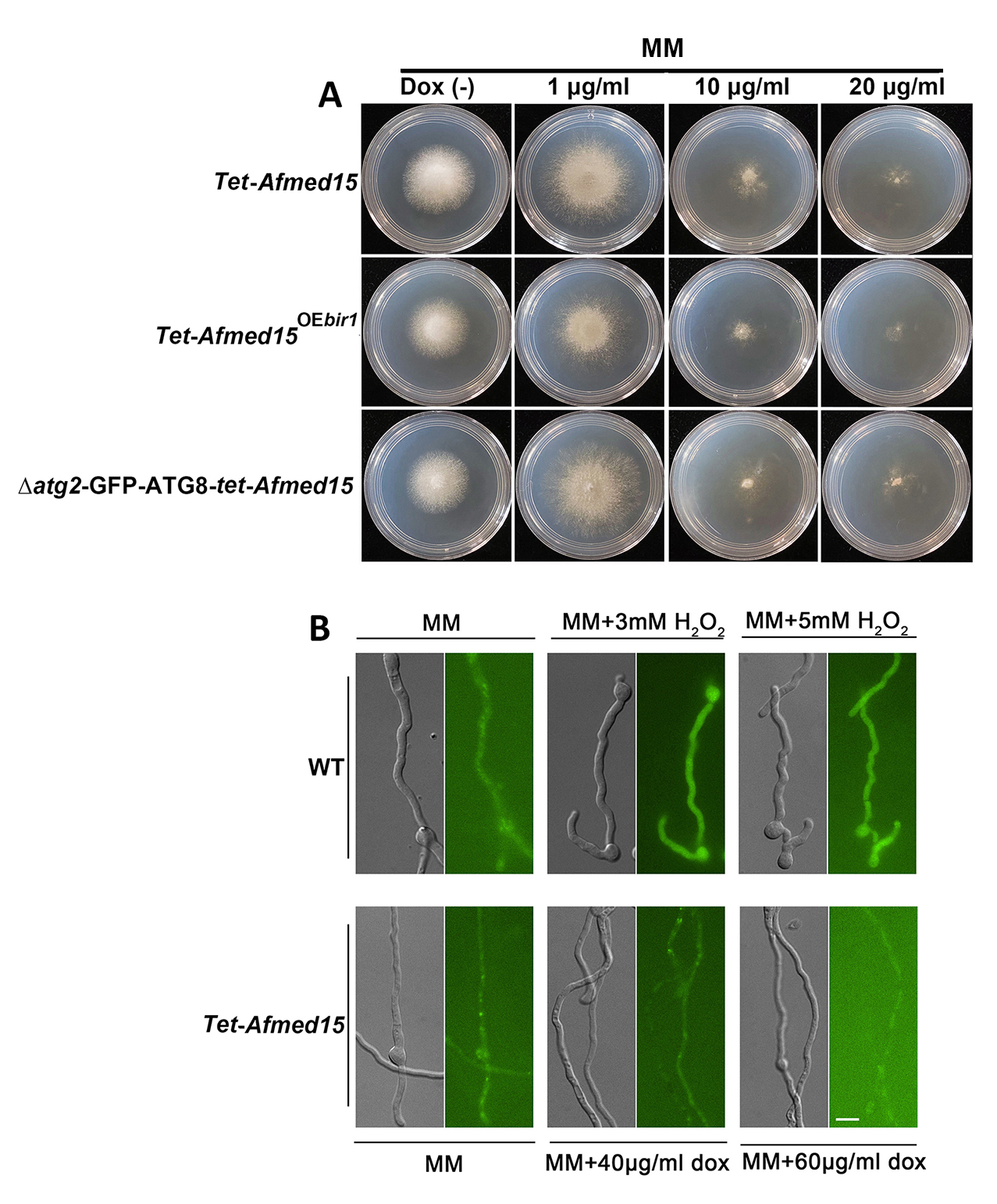

Supplement: FIG S4 [file mSphere.00771-20-sf004.tif]

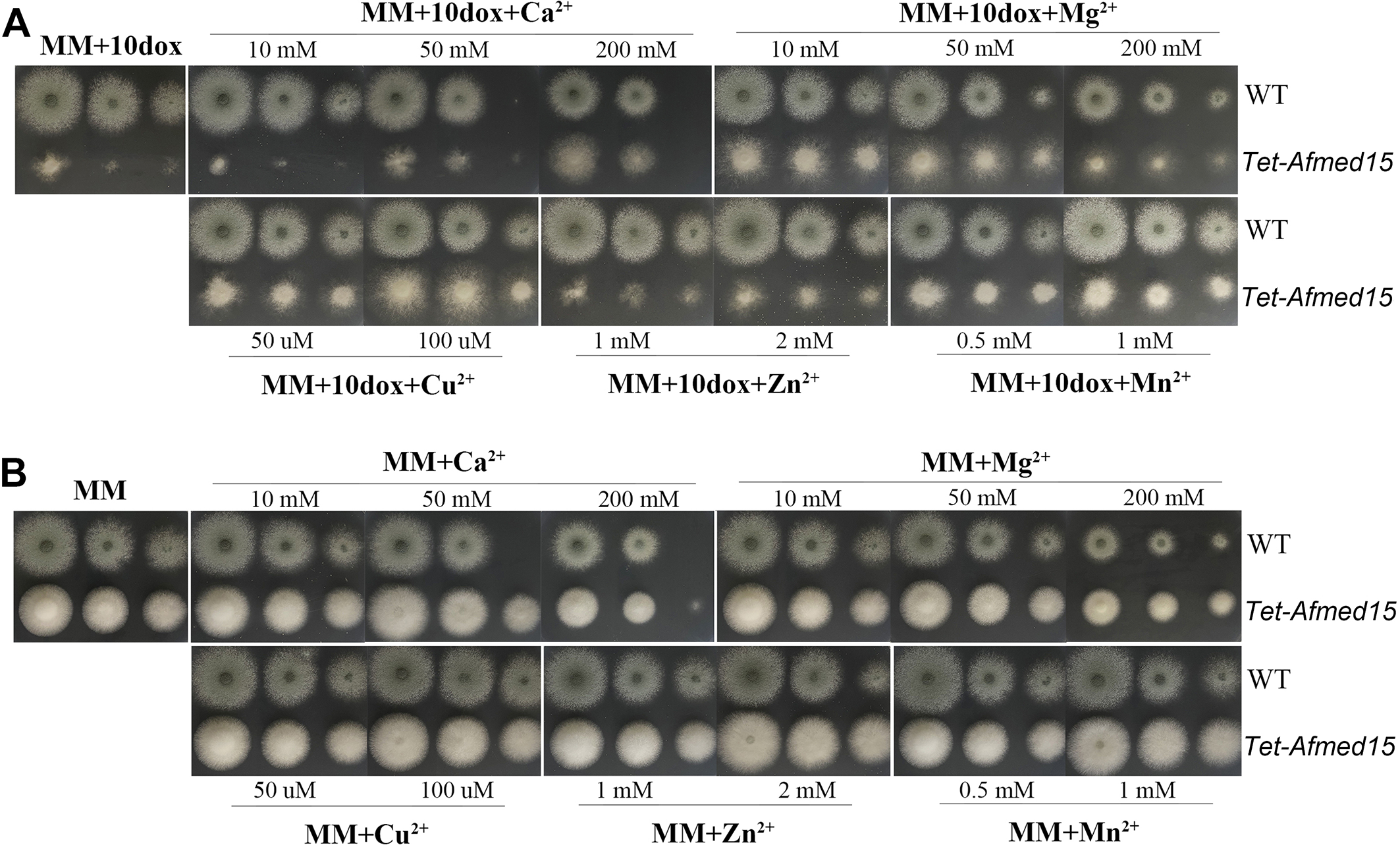

Supplement: FIG S5 [file mSphere.00771-20-sf005.tif]
