# Supplementary material for: Precise Expression of Afmed15 Is Crucial for Asexual Development, Virulence, and Survival of Aspergillus fumigatus
Source: mSphere. 2020 Oct 7;5(5):e00771-20. doi: 10.1128/mSphere.00771-20 (PMC7568654; doi:10.1128/mSphere.00771-20)
Supplement: TABLE S1 [file mSphere.00771-20-st001.docx]

| **strain** | **Genotype** | **Reference or Source** |
| --- | --- | --- |
| Af293 | Wild type | FGSC |
| T1033 | *Afmed15::T-DNA* | This study |
| A1160 | *Δku80;pyrG1* | FGSC |
| A1161 | *Δku80*; *A1160::pyrG1* | (1) |
| TN02A7 | *pyrG89;pyroA4;nkuA::argB2;riboB2* | (2) |
| *ΔAnmed15* | *ΔAnmed15::pyr4;pyrG89;pyroA4;nkuA::argB2; riboB2* | This study |
| *ΔAfmed15* | *ΔAfmed15::pyr4;Δku80,pyrG1* | This study |
| *Tet-Afmed15* | *Tet(p)::Afmed15::ptrA;Δku80*;*A1160*::*pyrG1* | This study |
| *ΔAfmed15^C^* | *ΔAfmed15::pyr4;Afmed15::hph;Δku80;pyrG1* | This study |
| *ΔAfmed15^OEbrlA^* | *ΔAfmed15::pyr4;gpd(P)::brlA::hph;Δku80; pyrG1* | This study |
| *ΔAfmed15^OEabaA^* | *ΔAfmed15::pyr4;gpd(p)::abaA::hph;Δku80; pyrG1* | This study |
| *ΔAfmed15^OEwetA^* | *ΔAfmed15::pyr4;gpd(p)::wetA::hph;Δku80; pyrG1* | This study |
| *Afmed15-GFP* | *Afmed15::GFP::pyr4;Δku80;pyrG1* | This study |
| GFP-Atg8-A1161 | *gpd(p)::gfp::atg8::hph;Δku80*;*A1160*::*pyrG1* | This study |
| GFP-Atg8-*tet-Afmed15* | *tet(p)::Afmed15::ptrA; gpd(p)::gfp::atg8::hph;Δku80*;*A1160*::*pyrG1* | This study |
| *Δatg2-*GFP-Atg8-A1161 | *Δatg2::phle;gpd(p)::gfp::atg8::hph;Δku80*; *A1160*::*pyrG1* | This study |
| *Δatg2**-*GFP-Atg8-*Tet-Afmed15* | *Δatg2::phle;Tet(p)::Afmed15::ptrA; gpd(p)::gfp::atg8::hph;Δku80*; *A1160*::*pyrG1* | This study |
| *Tet-Afmed15^OEbir1^* | *Tet(p)::Afmed15::ptrA;gpd(p)::bir1::hph;Δku80*; *A1160*::*pyrG1* | This study |

Table S1 strains used in this study

1. Jiang H, Shen Y, Liu W, Lu L. 2014. Deletion of the putative stretch-activated ion channel Mid1 is hypervirulent in Aspergillus fumigatus. Fungal Genet Biol 62:62-70.

2. Nayak T, Szewczyk E, Oakley CE, Osmani A, Ukil L, Murray SL, Hynes MJ, Osmani SA, Oakley BR. 2006. A versatile and efficient gene-targeting system for Aspergillus nidulans. Genetics 172:1557-1566.
