# Supplementary material for: Precise Expression of Afmed15 Is Crucial for Asexual Development, Virulence, and Survival of Aspergillus fumigatus
Source: mSphere. 2020 Oct 7;5(5):e00771-20. doi: 10.1128/mSphere.00771-20 (PMC7568654; doi:10.1128/mSphere.00771-20)
Supplement: TABLE S2 [file mSphere.00771-20-st002.docx]

**TABLE S2** Primers used in this study

| Primer Name | Primer Sequence (5’-3’) | | | |
| --- | --- | --- | --- | --- |
| Afmed15-P1 | CATCCGCTCCCGTCAACTAA | | |  |
| Afmed15-P2 | GGCAGATAGGACTTGGGTAGAG | | |  |
| Afmed15-P3 | CGATTAAGTTGGGTAACGCCAGAAGCAGGCTCGAAGGCAAA | | |  |
| Afmed15-P4 | ATAAGTAGCCAGTTCCCGAAAGCATTAACGCACGGCCTGAGAA | | |  |
| Afmed15-P5 | CAGGACCGTCAATCGTATGG | | |  |
| Afmed15-P6 | CGCTGAACAAACCGACAAAA | | |  |
| Afmed15-SF | CAATACCTTACCCGAAAGAACA | | |  |
| Afmed15-SR | CGAGAAACTGAGGGCTGATAGAG | | |  |
| Anmed15-P1 | CTCGGCTGACTTGGCTCTC | | |  |
| Anmed15-P2 | CCGACCGGTGTCTTAAGACTTG | | |  |
| Anmed15-P3 | CGATTAAGTTGGGTAACGCCAGATCGAGGATAAGTCCGCGC | | |  |
| Anmed15-P4 | ATAAGTAGCCAGTTCCCGAAAGCGTGGAACACTAAGCGCTGAC | | |  |
| Anmed15-P5 | CGTTGCATGAGTTACGTCCAC | | |  |
| Anmed15-P6 | GACATGAGGACAACAAGCCAAC | | |  |
| Anmed15-SF | TCGAGCTACGAAATGCCGCAC | | |  |
| Anmed15-SR | GCAATGCGACAGACGTTCTC | | |  |
| Pyr4-F | TGGCGTTACCCAACTTAATCG | | |  |
| Pyr4-R | GCTTTCGGGAACTGGCTACTTAT | | |  |
| Pyr4 up | TGAACGGCATTACCAAGGAAG | | |  |
| Pyr4 down | TACAGCGGAAGGCAATCAAG | | |  |
| Med15^C^-F | GAGGTAATCCTTCTTTCTAGA GCTGCTATCGAGATGCTACC | | |  |
| Med15^C^-R | ACGACGGCCAGTGCCAAGCTTTCTAGCTGCGAAGTGCCATC | | |  |
| Med15 down | TCCGCCCTCCAACCGCTAAA | | |  |
| AD1 | wgtgnagwancanaga | | |  |
| AD2 | agwgnagwancawagg | | |  |
| AD3 | ngtcgaswganawgaa | | |  |
| AD4 | gtncgaswcanawgtt | | |  |
| RB1 | ggcactggccgtcgttttacaac | | |  |
| RB2 | aacgtcgtgactgggaaaaccct | | |  |
| RB3 | cccttcccaacagttgcgca | | |  |
| LB1 | gtgtaaagcctggggtgcctaatgagtg | | |  |
| LB2 | agctaactcacattaattgcgttgcg | | |  |
| LB3 | cggggagaggcggtttg | | |  |
| Afmed15-GFP P1 | GCAGCAGCTTCAACAACAGG | | | |
| Afmed15-GFP P2 | GAGTTCCACACGTTTACGGTC | | | |
| Afmed15-GFP P3 | CCAGCGCCTGCACCAGCTCCCATGGCATAGATGGCAATGC | | | |
| Afmed15-GFP P4 | CATCAGTGCCTCCTCTCAGACAGGGAAAGATGGCACTTCGCAGC | | | |
| Afmed15-GFP P5 | GAGAGTCGCAGTTCCGTACAC | | | |
| Afmed15-GFP P6 | CCGACGAACATCTGAACACTATC | | | |
| GFP+PyrG F | GGAGCTGGTGCAGGCGCTGG | | | |
| GFP+PyrG R | CTGTCTGAGAGGAGGCACTGATG | | | |
| GFP+PyrG up | GATACAGGTCTCGGTCCCTA | | | |
| GFP+PyrG down | GTGAAGAGCATTGTTTGAGGC | | | |
| M13 F | GTAAAACGACGGCCAGT | | | |
| M13 R | CAGGAAACAGCTATGAC | | | |
| tet-Afmed15 P1 | GCTGCTGAGAGTCTAAAACGGG | |  |  |
| tet-Afmed15 P2 | CAGGGCAGAGTGAGTGGGTC | |  |  |
| tet-Afmed15 P3 | CAAGAGGCCATCTAGGCCTCCGATGGTGGAGCAGCCTAAC | |  |  |
| tet-Afmed15 P4 | CCGCTTGAGCAGACATCACCGCAGCCATGAATCCCGCAAAC | |  |  |
| tet-Afmed15 P5 | CCTGGCAACGGAAGAAGTACG | |  |  |
| tet-Afmed15 P6 | CTGAAAGATCTGTCGCTGCTGC | |  |  |
| tet-Afmed15 SF | GAGGCCTAGATGGCCTCTTG | |  |  |
| tet-Afmed15 SR | GGTGATGTCTGCTCAAGCGG | |  |  |
| tet-Afmed15-P SF | CATCCACGCCACCAGACAGC | |  |  |
| tet-Afmed15-P SR | CGACAACAAAGGGATTCAGAGG | |  |  |
| Tet up | CTGGAGGCACAACTAACAAG | |  |  |
| Tet down | GGCGGTATGGAGCTGTCTGA | |  |  |
| tet-Afmed15-RT F | ATGAATCCGCAGCAGCGACA | |  |  |
| tet-Afmed15-RT R | GCTGGCCTGCTTCTTGGGAA | |  |  |
| Tublin-RT F | TTCCGTCCCGACAACTTCGT | |  |  |
| Tublin-RT R | CACAGCCTTCAGCCTCACG | |  |  |
| brlA RT-F | GGGCCATACGGAGTCGATTG | |  |  |
| brlA RT-R | GGCGAGTGCGTCTTGAAGGT | |  |  |
| abaA RT-F | GACTGGCAGCCCGAGTGTATT | |  |  |
| abaA RT-R | GTCATCACGACCACTCATCCC | |  |  |
| wetA RT-F | CCCATCAGTCACCGCAACC | |  |  |
| wetA RT-R | GGGAGGAGTGGACGGTGAT | |  |  |
| Tublin-RT F | TTCCGTCCCGACAACTTCGT | |  |  |
| Tublin-RT R | CACAGCCTTCAGCCTCACG | |  |  |
| Gpd-GFP-Atg8-F | GGGCTGCAGGAATTCGATATCATGAGTAAAGGAGAAGAACTTTTCACTG |  |  |  |
| Gpd-GFP-Atg8-R | GGTATCGATAAGCTTGATATCTCAGCAGTCACCGAAAGTGTTC |  |  |  |
| ΔAtg2-P1 | TCGGCTCATTTGCGTCGGAAGA |  |  |  |
| ΔAtg2-P2 | GTACCAAGGCGGGTTACGAC |  |  |  |
| ΔAtg2-P3 | TAATCAATTGCCCGTCTGTCAACCCGTTTAGGACAAGCACC |  |  |  |
| ΔAtg2-P4 | GCTTACATTCACGCCCTCCTGCAACGGCAAACTGACACGA |  |  |  |
| ΔAtg2-P5 | CCAAAGAGCCCTGTCGATTA |  |  |  |
| ΔAtg2-P6 | TTTAGAGGCCGCCGACTTAC |  |  |  |
| ΔAtg2-SF | GCTCCCTTCTCACATTACGG |  |  |  |
| ΔAtg2-SR | TGTTGAGTCTTTCGGGCTGT |  |  |  |
| Phle-F | TGACAGACGGGCAATTGATTA |  |  |  |
| Phle-R | AGGAGGGCGTGAATGTAAGC |  |  |  |
| Phle up | CGAGTGGTCGGAGGTCGTGT |  |  |  |
| Phle down | CCATGACTTCCATCGTATGCC |  |  |  |
| gpd-BrlA F | tttatgtttagatccactagtATGGCGTACGGGTGTAAGTCTG |  |  |  |
| gpd-BrlA R | gcatagtaccgagaaactagtTTACTCATCCCATTCCATACTGATCT |  |  |  |
| gpd-AbaA F | tttatgtttagatccactagtATGGCTACTGACTGGCAGCC |  |  |  |
| gpd-AbaA R | gcatagtaccgagaaactagtTCATTGGACCGCCTCAGTTG |  |  |  |
| gpd-WetA F | tttatgtttagatccactagtATGTTCGCTCAACCATTCGATC |  |  |  |
| gpd-WetA R | gcatagtaccgagaaactagtTTAGCAGAGGACAGCCTCCAGT |  |  |  |
| Gpd-BIR1 F | gggctgcaggaattcgatatcACTGATCTGGCGCAGTCTTGC |  |  |  |
| Gpd-BIR1 R | ggtatcgataagcttgatatcTCAATCAATGCACTCGATCCC |  |  |  |
| OE BIR q-PCR F | GGGTGGGAGCCGGAGGATAA |  |  |  |
| OE BIR q-PCR R | TGCCATCGTGTGGCCATTGA |  |  |  |
| hph-up-SpeⅠ | CGGACTAGTGAATTCCCTTGTATCTCTAC |  |  |  |
| hph-down-SpeI | CGGACTAGTTCGAGTGGAGATGTGGA |  |  |  |
